# Characterization and transformation of *TtMYB1* transcription factor from *Tritipyrum* to improve salt tolerance in wheat

**DOI:** 10.1186/s12864-024-10051-5

**Published:** 2024-02-09

**Authors:** Yuanhang Mu, Luxi Shi, Huan Tian, Huaizhi Tian, Jv Zhang, Fusheng Zhao, Qingqin Zhang, Suqin Zhang, Guangdong Geng

**Affiliations:** 1 College of Agriculture, Guizhou University, Guiyang, Guizhou China; 2 Zunyi Acadamy of Agricultural Sciences, Zunyi, Guizhou China; 3 Guizhou Subcenter of National Wheat Improvement Center, Guiyang, Guizhou China

**Keywords:** *Tritipyrum*, *TtMYB1*, Transformation, Phenotype, Physiology, Salt tolerance, Interacting protein

## Abstract

**Background:**

Common wheat (*Triticum aestivum* L*.*) is a worldwide cereal crop, which is an integral part of the diets of many countries. In addition, the *MYB* gene of wheat plays a role in the response to salt stress.

**Results:**

“Y1805” is a* Tritipyrum *variety that is relatively tolerant to salt. We used transcriptome analysis to show that the “Y1805” *MYB* gene was both highly expressed and sensitive to salt stress. Compared with control roots, the level of *MYB *expression during salt stress was higher, which rapidly decreased to control levels during the recovery process. *MYB* gene relative expression showed the highest levels in “Y1805” roots during salt stress, with the stems and then leaves being the next highest stressed tissues. The novel *MYB* gene (*TtMYB1*) was successfully cloned from “Y1805”. It showed a coding sequence length of 783 bp with 95.79% homology with *Tel2E01G633100* from* Thinopyrum elongatum*. TtMYB1 and MYB from *Th. elongatum* were clustered in the same branch using phylogenetic analysis, which indicated high similarities. The *TtMYB1* gene is located in the nucleus. The coleoptile method was employed when a *TtMYB1* overexpression vector was used during transformation into “1718” (common wheat). Under high salt stress, *TtMYB1* leaves of overexpression lines had decreased wilting, when compared with wild-type (WT) plants. During normal conditions, salt stress, and recovery, the lengths of the roots and the heights of seedlings from the overexpression lines were found to be significantly greater than roots and seedlings of WT plants. In addition, during high salt stress, the overexpression lines showed that proline and soluble sugar levels were higher than that of WT plants, but with lower malondialdehyde levels. Forty-three proteins that interacted with TtMYB1 were identified using the yeast two-hybrid assay. Protein-protein interaction analyses indicated that most were SANT domain-containing and Wd repeat region domain-containing proteins. Among these proteins, ribosomal proteins were the main node. Abiotic stress-related terms (such as “carbonate dehydratase activity”, “protein targeting peroxisomes”, and “glutathione peroxidase activity”) were enriched in GO analysis. In KEGG analysis, “carbohydrate metabolism”, “environmental information processing”, “genetic information processing”, “signaling and cell precursors”, and “energy metabolism” pathways were enriched.

**Conclusion:**

The *TtMYB1* gene might enhance salt tolerance by increasing proline and soluble sugar content and antioxidase activity in transgenic wheat. It therefore has the potential to enhance high salt tolerance in plants.

**Supplementary Information:**

The online version contains supplementary material available at 10.1186/s12864-024-10051-5.

## Introduction

Common wheat (*Triticum aestivum* L.) is a worldwide cereal crop, which is an integral part of the diets of many countries [1]. Soil salinization is a common factor that reduces crop growth and yield in modern agriculture [2, 3]. To cope with saline-alkaline stress, plants have evolved sophisticated regulatory mechanisms, including those involved in signal recognition and transduction, transcriptional regulation, and responses to high salt stress [4, 5]. It is therefore crucial to identify important genes for breeding crops resistant to high salt conditions.

MYB transcription factors (TFs) are only found in eukaryotes, comprising a large and diverse family of plant TFs. The TFs are involved in many essential physiological and biochemical processes [6, 7]. MYB TFs from plants have an N-terminal domain and MYB domain of approximately 51–52 amino acids. The repeats of MYB are characterized by a hydrophobic core that contains a total of three conserved tryptophan residues [8]. A conserved DNA-binding domain is shared in MYB proteins, which is comprised of 1 − 4 partially nonhomologous amino acid sequence repeats. When considering the adjacent repeats, the plant proteins of MYB in plants are divided into four distinct groups: MYB-related (MYBR, which contains one R1 or the R2-like repeat, only), R2R3-MYB (with two R2/3R similar repeats), 3R-MYB (with three R1/R2/R3-like repeats), and atypical MYB proteins (4R-MYB, with four R1/R2-like repeats; similar to CDC5) [9].

The salt stress response is at least partially controlled by the MYB family [10]. During high salt stress of plants, R2R3-MYB TFs mainly respond by participating in the abscisic acid (ABA) signaling pathway, regulating the levels of reactive oxygen species (ROS), and improving osmotic stress resistance [11, 12]. When there is overexpression of *Arabidopsis*, it can result in decreases in the levels of ABA-dependent gene transcripts during stress from high salt conditions [13]. When *TaMYB86B* is overexpressed, there is an increase of wheat to high salt tolerance. This process involves the regulation of ion homeostasis, maintainance in osmotic balance, and a decrease in ROS levels [14]. In rice, it was confirmed that the R2R3-type MYB gene, *OsMYB91*, was involved in tolerance to stress from high salt and in plant growth [15]. Overexpression of the *SlMYB102* gene helps to accumulate a large number of ROS scavengers, slow the rate of ROS production, and thereby improve tomato tolerance to high salt stress [16]. An increase in the expression of *MdMYB108L* has been shown to be involved in increases of photosynthesis capacity of hairy root tissues (leaves) during salt stress, and it has also been shown that when bound to the salt-tolerant *MdNHX1* promoter, it results in positive transcription in apples, to increase high salt tolerance in transgenic plants [17].

It has been reported that there was a diverse range of MYB TF functions in different plant species [8, 9]. Salt and polyethylene glycol significantly induce the expression of *GhMYB108*-*like* in upland cotton [18]. When *AmMYB1* TF is constitutively expressed in transgenic tobacco plants in salt-tolerant *Avicennia marina*, it is more resistant to high salt stress [19]. G*hMYB36* is a gene that encodes a R2R3-type MYB protein in *Gossypium hirsutum*. It results in tolerance to drought and wilt resistance to *Verticillium* wilt in *Arabidopsis* and cotton [20]. The maintainance of Fe homeostasis is important when *Arabidopsis* responds to stress from ammonia, which has been correlated with the expressions of *MYB28* and *MYB29* [21]. Z*mMYB31*, an R2R3‑MYB TF in maize, is an important positive regulator of cold and peroxide stress. It is involved in the regulation of expression of genes involved in stress due to chilling, to decrease the levels of ion extravasation, the content of ROS, and low temperature photoinhibition, to improve resistance to low temperature [22]. Drought and cold induce expression of *ApMYB77*, and overexpression in *Arabidopsis* has been shown to enhance tolerance to freezing [23]. *MYB59* plays a role in the responses to stress and plant growth as a negative regulator of homeostasis and calcium signaling [24]. *AtMYB96* is also involved in pathogen resistance via a mechanism inducing salicylic acid biosynthesis in *Arabidopsis*. Although they improve abiotic stress tolerance and pathogen resistance, plant R2R3-MYB TFs are also involved in defensive responses to insects. In wheat, *TaMYB19*, *TaMYB29*, and *TaMYB44* have roles as co-regulators of phloem-based defenses against the English grain aphid [25].

In *Triticeae*, E genome species (e.g., halophile wheatgrass *Thinopyrum elongatum*) is an important gene pool involved in wheat genetic improvement. It has genetic variations in many important agronomic traits, such as salt tolerance, cold tolerance, and disease resistance [26]. *Tritipyrum* displays salt tolerance and is derived from crosses of *Triticum aestivum* and *Th. elongatum* [27]. In addition, gene families of the *MYB* gene have been found in many plant species, including common wheat, rice, maize, tomato, apple, and *Arabidopsis* [16, 17, 22, 23, 25]. Nonetheless, the studies of the *MYB* gene from the salt-tolerant *Tritipyrum* have not been reported. In the current study, *TtMYB1*, an R2R3-type *MYB* gene, was cloned from *Tritipyrum*, to characterize its sequence, evolutionary relationships, patterns of expression, gene functions, and the interactions of proteins during high salt stress. The present study therefore revealed an increased understanding of the salt-tolerant mechanism of *MYB* genes, and provides the basis for the breeding of more salt-tolerant wheat varieties.

## Results

### MYB gene screening by transcriptomic analysis

Transcriptome analysis was carried out on the roots of salt-tolerant *Tritipyrum* “Y1805” and salt-sensitive wheat “Chinese spring” (“CS”) after 5 h salt stress and 1 h recovery to select important genes in the response to salt stress. The relative expression level [log_2_fold change (log_2_FC) = 6.82] of *Tel2E01G633100* in *Tritipyrum* “Y1805” was significantly higher than in wheat “CS” under salt stress (Table 1). However, this gene showed no change in expression after recovery in “Y1805”. Basic local alignment search tool (BLAST) analysis indicates that *Tel2E01G633100* contained two SANT domains, therefore it might be an MYB protein. *Tel2E01G633100* was annotated and assigned to the gibberellic acid mediated signaling pathway (GO:0009740) and gibberellin biosynthetic process (GO:0009686) by GO analysis. Gibberellin can promote the elongation and growth of roots to enhance its water absorption capacity. Therefore, “Y1805” specific *Tel2E01G633100* might be an important gene involved in the salt stress response.

**Table 1:** Relative expression level (log_2_FC) of *Tel2E01G633100* under salt stress and recovery conditions in two wheat varieties

Materials	Salt stress	Recovery
“Y1805”	6.82	NE
“CS”	NE	NE

“NE” indicates no expression

### Expression patterns of the MYB gene in Y1805

**Fig. 1:**
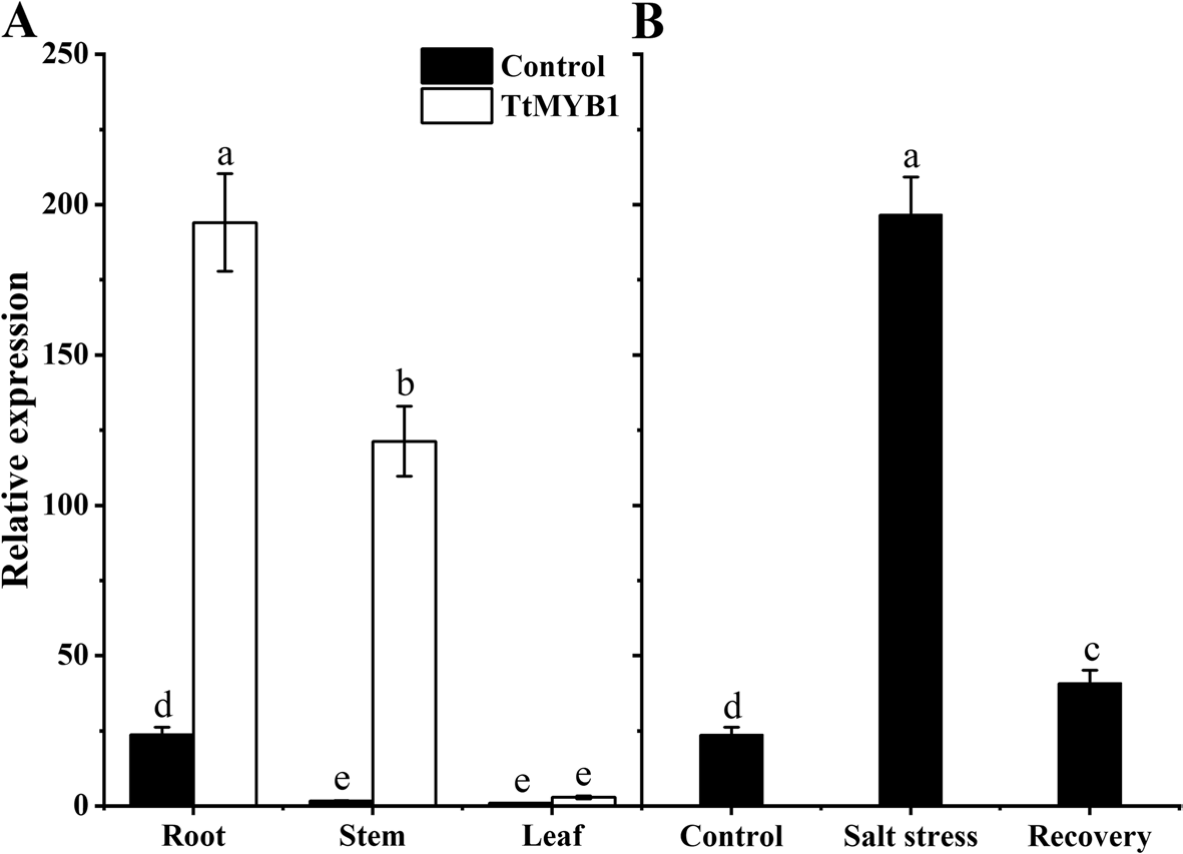
Expression levels of *MYB* in “Y1805” measured by qPCR analysis. **A** Relative expression levels of the *MYB* in roots, stems, and leaves under salt stress. **B** Relative expression levels of *MYB* in roots under salt stress and in recovery. Bars indicate means with SDs (*n* = 3). Values with different letters are significantly different at *p* < 0.05

To investigate the spatial and temporal expression pattern of *MYB*, we analyzed the expression levels of *MYB* in roots under salt stress and recovery conditions and in various tissues using quantitative real-time PCR (qPCR) analysis. The relative expression level of the *MYB* gene was the highest under salt stress in the roots of “Y1805”, stems were the next highest, and then leaves (Fig. 1A). The expression level of the *MYB* gene in the roots was 1.76 and 13.06-fold higher than those of stems and leaves, respectively. In addition, the *MYB* expression level was significantly (7.22-fold) higher than the control under salt stress in the roots. However, it dropped rapidly after recovery (Fig. 1B). These results were consistent with the above transcriptome data, indicating that “Y1805”-specific *MYB* were expressed highly and sensitively in the roots under salt stress to adapt.

### *TtMYB1* cloning and sequence analysis

**Fig. 2:**
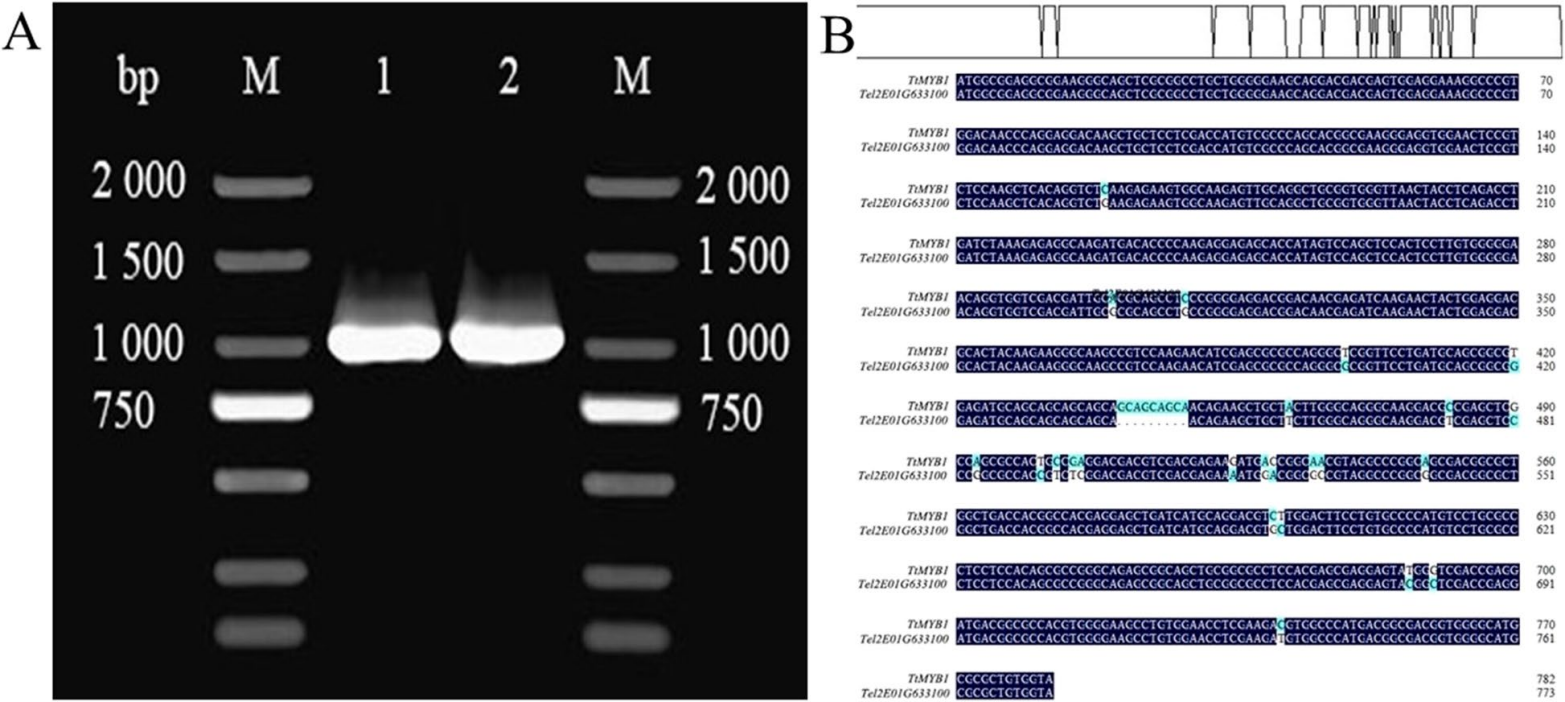
Cloning and alignment of *TtMYB1* from *Tritipyrum* “Y1805”. Amplified band with “Y1805” cDNA as a template. 1, 2: Amplified bands; M, 2000 bp DNA marker. **B** Alignment of *TtMYB1* gene from *Tritipyrum* “Y1805” and *Tel2E01G633100* gene from *Th. elongatum*. Blue gaps indicate different bases

**Fig. 3:**
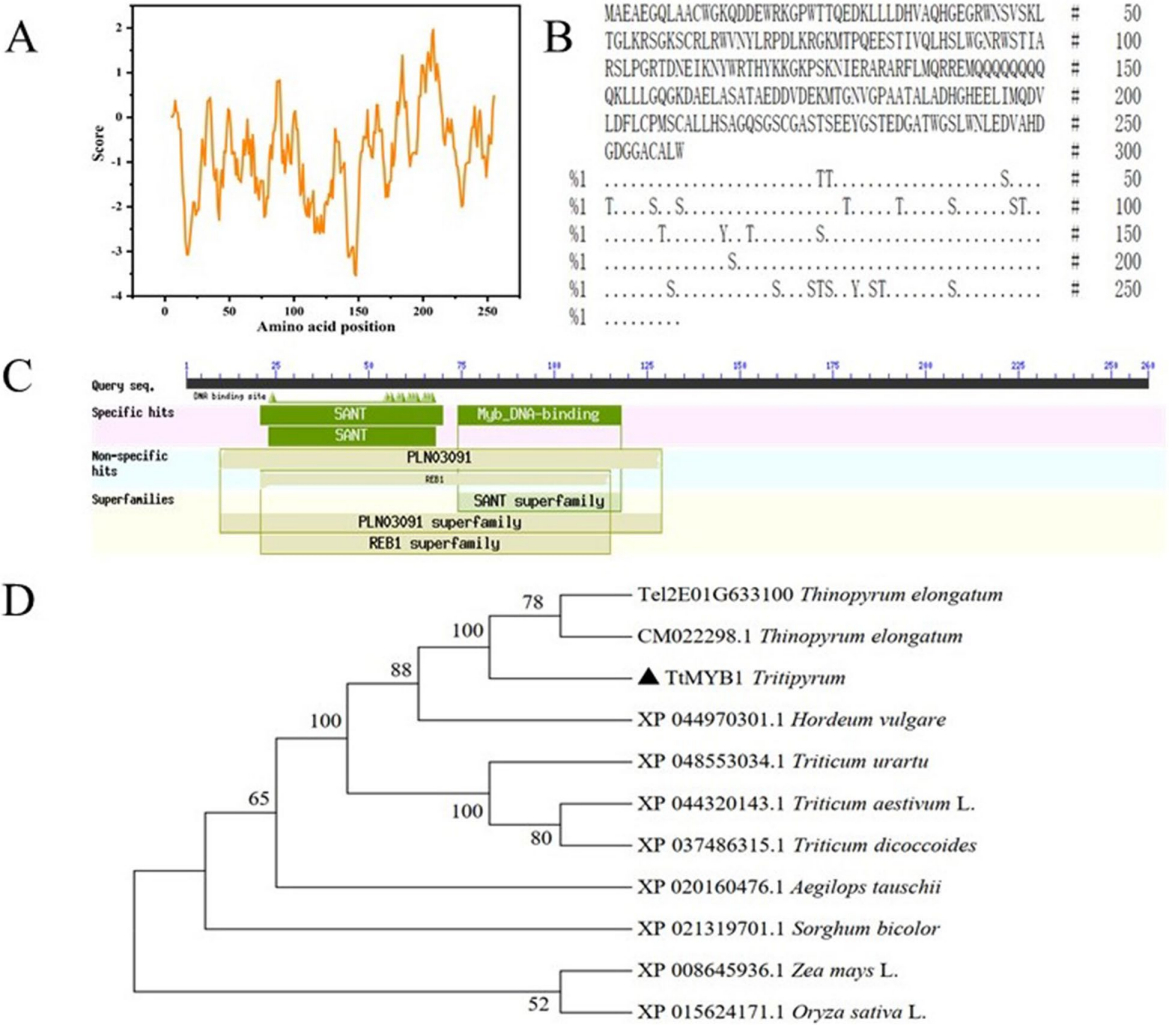
Bioinformatics analysis of TtMYB1 protein. **A** Hydrophilicity. **B** Protein phosphorylation sites. **C** Protein domain. **D** Phylogenetic tree of MYB proteins in various plant species

Using *Tel2E01G633100* specific primers, a 783 bp cDNA fragment corresponding to *Tel2E01G633100* was amplified from *Tritipyrum* “Y1805” by PCR (Fig. 2A; Supplementary Fig. S1) and named *TtMYB1* (GenBank accession no. SUB13704642 TtMYB1 OR344773). The *TtMYB1* sequence had 95.79% identity to *Tel2E01G633100*, with only 27 bp nucleotide changes between them (Fig. 2B). Therefore, *TtMYB1* was similar to *Tel2E01G633100* according to their cDNA sequences. In bioinformatics analysis, *TtMYB1* encoded 259 amino acids (aa). The TtMYB1 protein contained ten serine, three threonine, and two tyrosine residues, which could be protein kinase phosphorylation sites (Fig. 3A). Hydropathy plots predicted that TtMYB1 was predominantly hydrophilic, indicating it might play a role in plant salt stress tolerance (Fig. 3B). The TtMYB1 protein had SANT conserved domains at 20–70 aa and 73–121 aa in a region located at the N-terminal (Fig. 3C), indicating that TtMYB1 belongs to the 2R3R-type MYB transcription factor, and the domains might participate in the formation of a homo/heterodimer and regulate of transcription, respectively.

The phylogenetic tree of wheat MYB protein was constructed, and TtMYB1 and Tel2E01G633100 were grouped together (Fig. 3D). TtMYB1 and MYB from *Th. elongatum* were clustered in the same branch, which indicates they have high similarity. Therefore, we speculated that TtMYB1 of *Tritipyrum* might be similar to that of *Th. elongatum* in evolution and function.

### Homology modeling and molecular simulation of the TtMYB1 protein

**Fig. 4:**
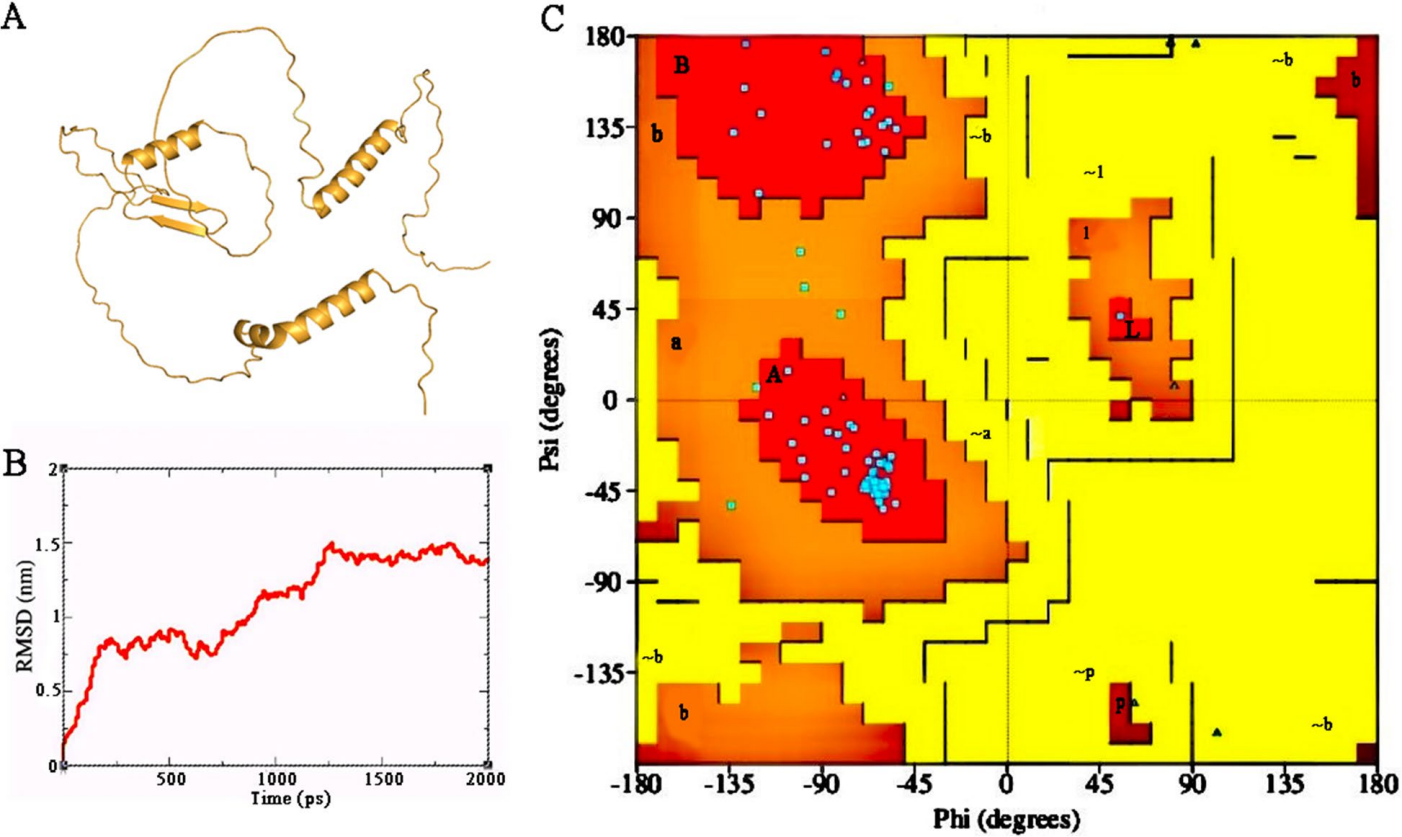
Homology modeling and molecular simulation of the TtMYB1 protein. **A** Homology modeling of the TtMYB1 protein. **B** Molecular dynamics simulation. Backbone of root mean squared deviation (RMSD) plotted versus time in picoseconds (ps). **C** Ramachandran plot analysis. A, B, and L regions: most favored residues; a, b, l, and p regions: additional allowed residues; ~a, ~b, ~l, and ~ p regions: generously allowed residues

Based on the Modeler 9.22 model, the N and C-terminal regions of the TtMYB1 protein had an α-helix and some reverse parallel β-chains (Fig. 4A). The compatibility between the model and c6kksA is 81.8%, indicating that the model is of good quality. The root mean square deviation (RMSD) analysis of the TtMYB1 protein backbone reached a peak (1.5312 nm) at 1257 picoseconds (ps), and the RMSD curve reached equilibrium after 1250 ps with a fluctuation range of 0.80–1.53 nm (Fig. 4B). The Ramachandran plot of the protein showed that the conformation was accurate. Because the model was accurate in over 90% of its most favorable regions, which are indicated in red [A, B, L], it could be considered that the protein model conformed to the rules of stereochemistry. The favorable region of the protein model in this study reached 92.6%, allowing for 7.4% of the amino acid residues in the residual region, and no residues in the disallowed region (Fig. 4C). These results indicate that the TtMYB1 protein structure was stable.

### Subcellular localization of TtMYB1

**Fig. 5:**
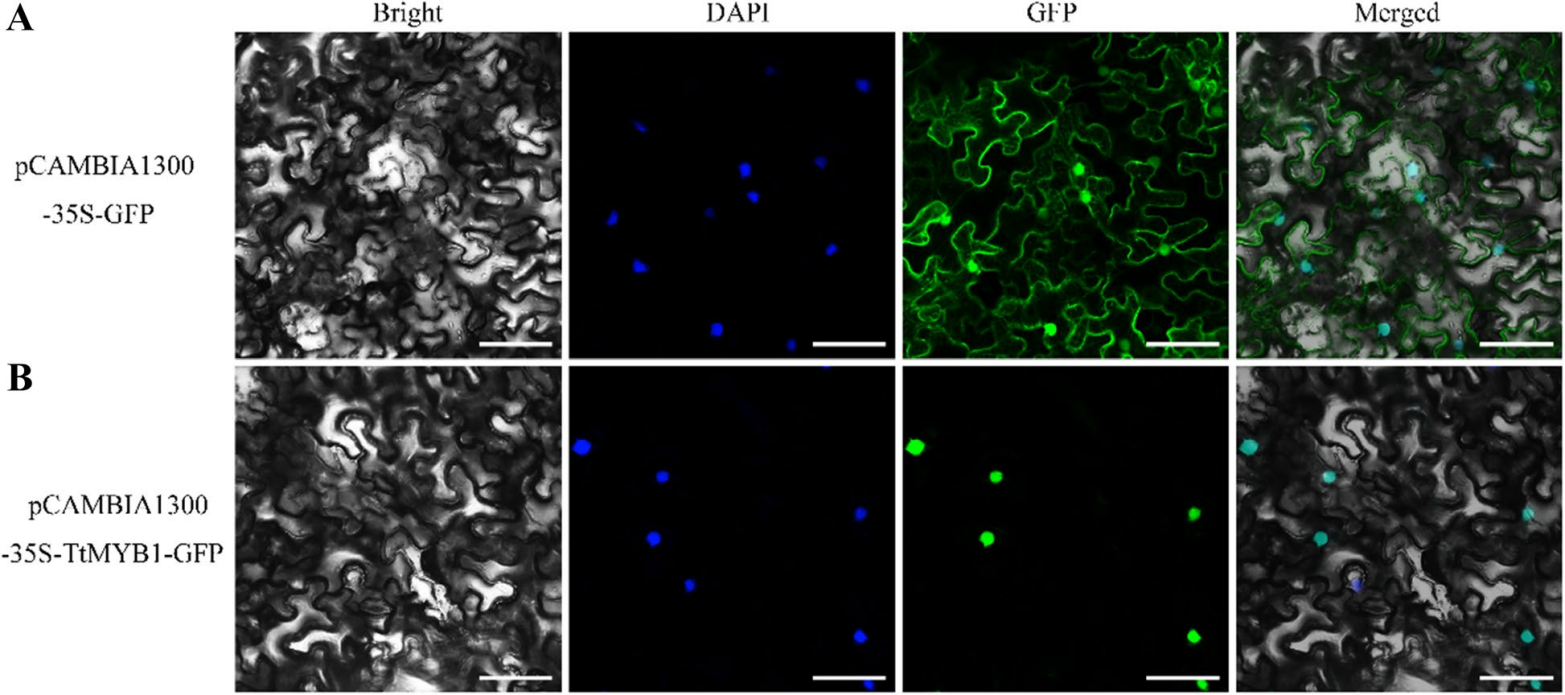
Subcellular localization of the TtMYB1 protein. **A** The vector controlling 1300-GFP was transformed into tobacco mesophyll cells. **B** Fusion protein construct 1300-TtMYB1-GFP was introduced into tobacco mesophyll cells. The sample was observed under a confocal laser-scanning microscope. Bright-field phase-contrast images, nuclear fluorescence (blue), GFP fluorescence (green), and merged images (green and blue) are shown. Scale bar = 20 μm

To investigate the subcellular location of the TtMYB1 proteins, a fusion protein transiently expressing 1300-*TtMYB1*-green fluorescence protein (GFP) was produced in tobacco mesophyll cells. The fluorescence emitted by the fusion protein was in the nucleus, overlapping the blue 4′,6-diamidino-2-phenylindole (DAPI) signal (Fig. 5), which means that *TtMYB1* is located in the nucleus. The results indicated that *TtMYB1* might contribute to transcriptional regulation.

### Coleoptile transformation in common wheat

**Fig. 6:**
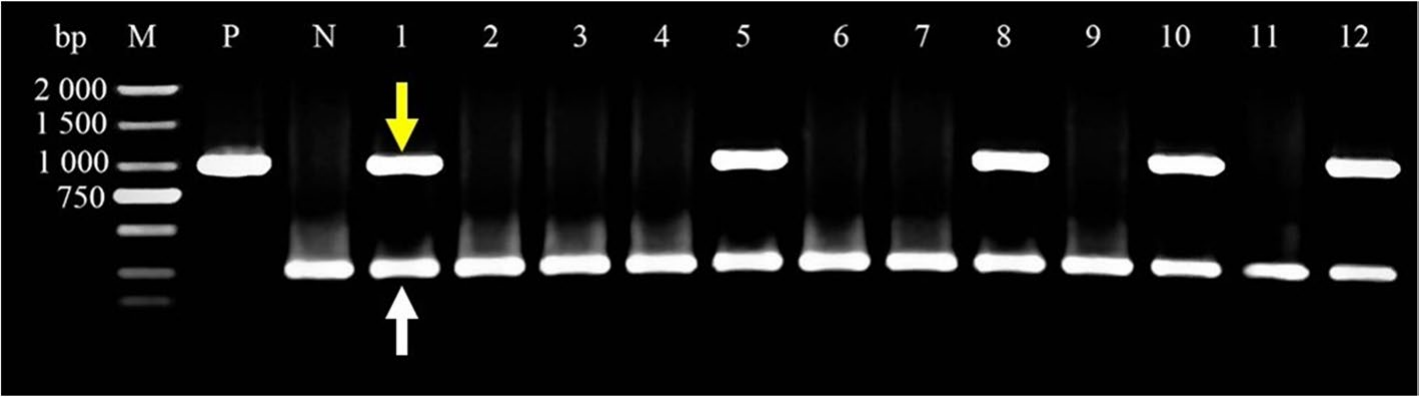
PCR confirmation of transgenic wheat harboring the *TtMYB1* gene. PCR detection of the *TtMYB1* gene in the genomic DNA of putative transgenic T_0_ wheat leaves. The fragment of the amplified *TtMYB1* gene is indicated by a yellow arrow. A white arrow shows the band of the amplified housekeeping *18 S* gene. M, 2,000 bp DNA marker; lanes 1, 5, 8, 10, and 12, transgenic plants; lanes 2, 3, 4, 6, 7, 9, and 11, non-transformed plants; P, positive control (*TtMYB1* recombinant plasmid); N, negative control (wild-type DNA)

To determine *TtMYB1* function, *TtMYB1* was transformed and overexpressed in common wheat. The coleoptile tips of common wheat were cut to expose the meristem and infected with *Agrobacteria* containing the *TtMYB1* gene. Transgenic-positive plants were confirmed by multiplex PCR using *TtMYB1*-specific primers and housekeeping *18 S* gene primers. The housekeeping primers amplified in all plant samples indicate that the DNA was of good quality (Fig. 6; Supplementary Fig. S2). Lanes 1, 5, 8, 10, and 12 show amplification with construct-specific primer sets, indicating these plants had incorporated the transgene.

### Overexpression of *TtMYB1* enhanced salt tolerance in wheat

#### Effect of salt stress on plant growth

**Fig. 7:**
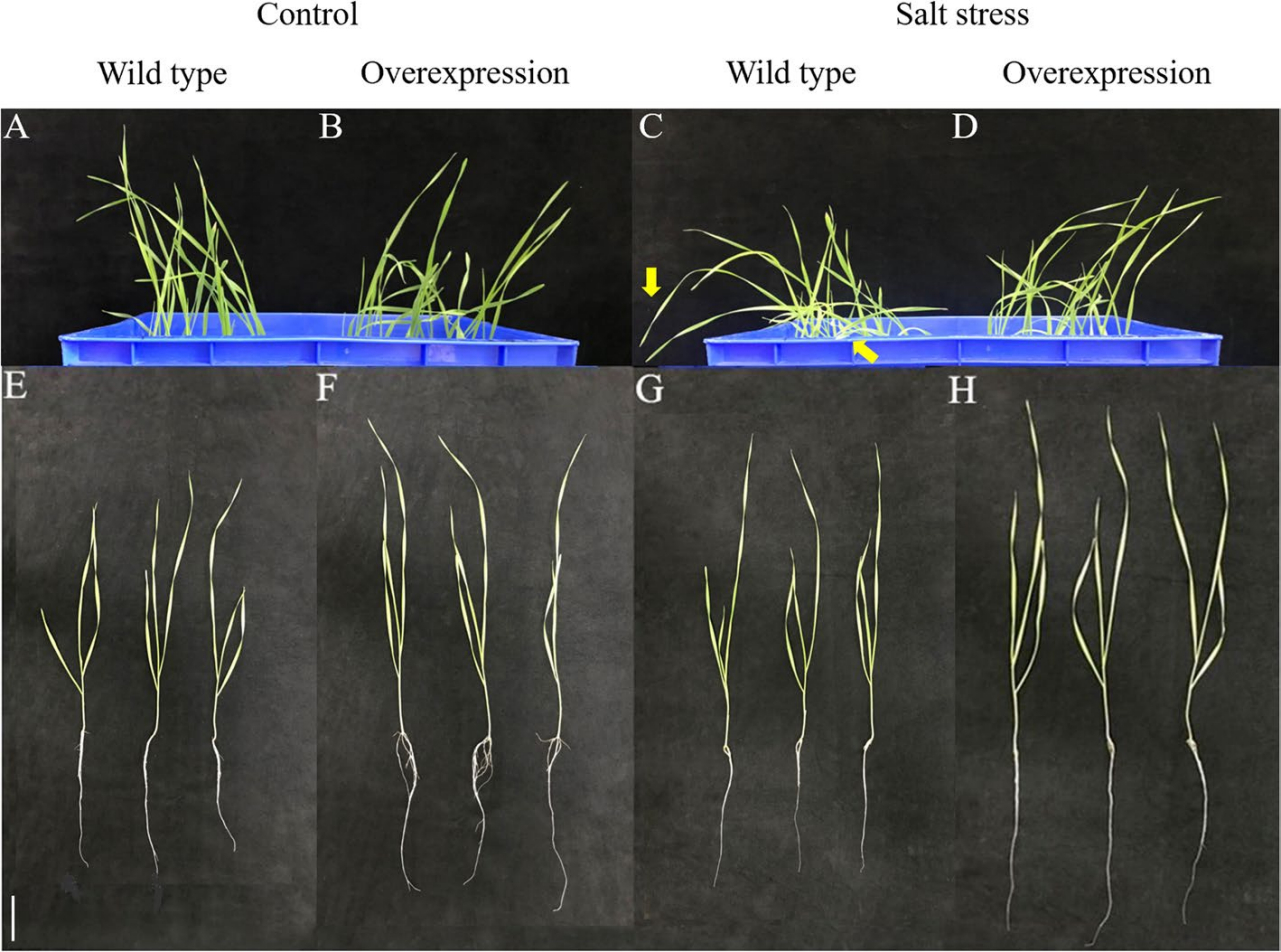
Effects of salt stress on the growth of *TtMYB1* overexpression wheat lines. **A**, **C**, **E**, and **G** wild-type wheat under normal and salt stress conditions. **B**, **D**, **F**, and **H** The overexpression line under normal and salt stress conditions. Arrows indicate severe wilt. Scale bar = 5 μm

Under NaCl stress, the leaves of *TtMYB1*-overexpressing wheat lines showed less wilting compared to these of the wild-type (WT) plants (Fig. 7A-D). Additionally, the overexpression lines exhibited a higher height and longer roots than the WT plants (Fig. 7E–H), indicating that the *TtMYB1* gene conferred wheat with salt tolerance.

**Fig. 8:**
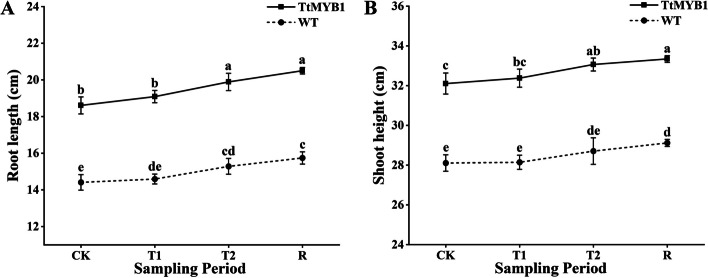
Effect of salt stress and recovery on plant growth. **A**, **B** Effects on wheat root length and shoot height. CK, T1, T2, and R represent the control, 5 h after salt stress, 24 h after salt stress, and 1 h after recovery, respectively. The column represents the mean value of SD (*n* = 3) and values with different letters are significantly different at *p* < 0.05

Under normal, salt stress, and recovery conditions, the root length and seedling height of the overexpression lines were significantly greater than those of WT plants (Fig. 8). This data suggested that overexpression of *TtMYB1* improved plant growth and salt stress had a greater inhibitory effect on the growth of the WT plants compared to the overexpression lines at the seedling stage.

### Effects of salt stress on physiological indicators

**Fig. 9:**
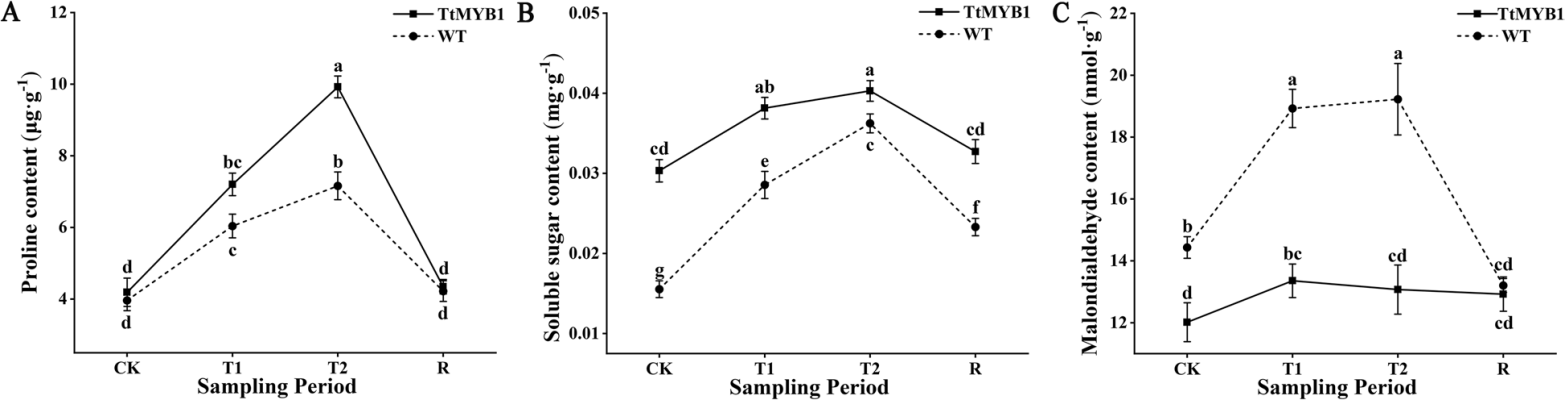
Effects of salt stress and recovery on osmotic regulation substances and MDA in wheat. **A**, **B**, and **C** The proline, soluble sugar, and MDA content in wheat. CK, T1, T2, and R represent the control, 5 h after salt stress, 24 h after salt stress, and 1 h after recovery, respectively. The column represents the mean value of SD (*n* = 3) and values with different letters are significantly different at *p* < 0.05

Proline accumulation is a common response to various abiotic stresses. It acts as an osmoprotectant, helping to protect cell membranes and proteins under stress [28, 29]. Under normal growth conditions, the proline content of the overexpression lines was slightly higher than that of the WT plants (Fig. 9A). After salt stress, the proline content of both the WT plants and overexpression lines significantly increased. With the extension of salt stress, the proline content in the overexpression lines increased rapidly, reached a peak at the T2 stage, and was significantly higher than that of the WT plants. After recovery, the proline content of the overexpression lines quickly dropped to the control level. These results indicated that the overexpression lines could respond more sensitively to salt stress to adapt quickly.

Soluble sugars act as osmotic substances and molecular chaperones to protect the structural and protein integrity and enhance enzyme activity, thus improving the resistance to abiotic stress [30]. Under normal, salt stress, and recovery conditions, the soluble sugar content of the overexpression line was significantly higher than that of the WT plant (Fig. 9B).

When plants are subjected to various abiotic stresses, the activity of ROS increases, followed by the production of membrane lipid peroxidation product MDA, which damages plant cells and can lead to cell death in severe cases [31, 32]. Compared with the WT plants, the overexpression lines showed a significant decrease in MDA content under salt stress (Fig. 9C). The MDA content of the WT plants rapidly increased with the extension of salt stress and reached a peak at the T2 stage, which was 1.54-fold that of the overexpression lines. The degree of increase in MDA content in the overexpression lines was significantly less than that of WT plants under salt stress. This might be attributed to salt tolerance in the lines overexpressing the *TtMYB1* gene.

### Interacting protein analysis of TtMYB1

**Fig. 10:**
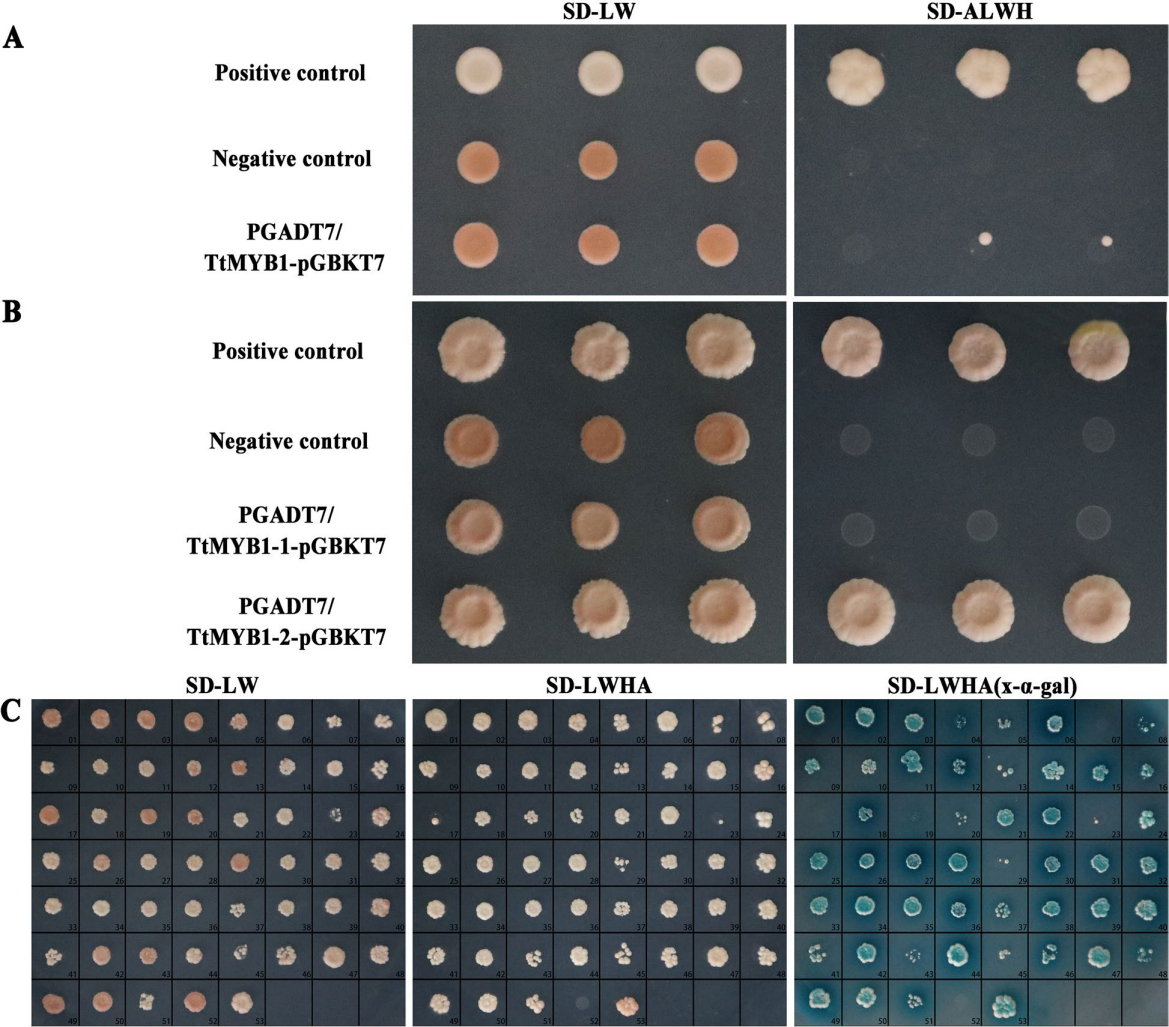
Identification of non-self-activating TtMYB1 protein and rotational validation of TtMYB1-1 interacting proteins. The SD-LW and SD-ALWH plate had aseptic spots, indicating that the TtMYB1 protein was toxic. SD-LW plates and SD-ALWH plates had plaques, indicating that the TtMYB1 protein had self-activation activity. SD-LW plates had white plaques and SD-ALWH plates had sterile plaques, indicating that the TtMYB1 protein had no self-activating activity. 1–51: 51 positive clones were identified. 52: Negative control (pGBKT7-laminC). 53: Positive control (pGBKT7-p53)

A yeast two-hybrid (Y2H) experiment was used to explore the proteins that interacted with TtMYB1 to enable a better understanding of the regulatory mechanism. The detection of self-activation showed that the control strains could grow normally on SD/-Leu/-Trp (SD-LW) plates, and three random colonies of pGADT7 + pGBKT7- *TtMYB1* transporters could grow on SD/-Ade/-His/-Leu/-Trp (SD-ALWH) plates (Fig. 10A). This indicated that the TtMYB1 protein was self-activating, and could not be used directly to screen the cDNA library. The *TtMYB1* was truncated into two fragments (*TtMYB1-1* and *TtMYB1-2*, Table S1) for further detection of self-activation. For TtMYB1-1, there were white plaques on the SD-LW plate and sterile plaques on the SD-ALWH plate, indicating that the TtMYB1-1 was a non-toxic and non-self-activating protein (Fig. 10B). Therefore, TtMYB1-1 was used to screen the library.

The library plasmid and the *TtMYB1-1*-pGBKT7 bait plasmid were co-transfected into Y2HGold yeast competent cells and spread on SD-LW, SD-ALWH plates, and SD-ALWH (x-α-gal) plates. The positive control and clones could grow normally on the SD-LW, SD-ALWH, and SD-ALWH (x-α-gal) plates. The negative control could grow normally on the SD-LW plate, but it could not grow on the SD-ALWH and SD-ALWH (x-α-gal) plates, because it did not activate histidine, adenine, and x- α-gal reporter genes. In total, 51 clones were positive. Rotational validation experiments further showed that all 51 proteins might interact with TtMYB1 (Fig. 10C). The sequencing data and statistical results of the TtMYB1-interacting proteins are shown in Table S2. Eight proteins (such as XM_044551351.1, XM_044583951.1, and XM_044597708.1) were repetitively identified according to the sequencing data and BLAST analysis. Therefore, 43 interacting proteins were obtained except repetitive proteins.

**Fig. 11:**
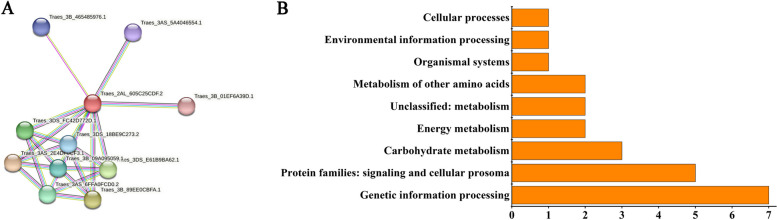
TtMYB1 interacting-protein analysis. **A** Protein-protein interacting analysis. **B** KEGG pathways of the TtMYB1 potential interacting proteins

Protein-protein interaction (PPI) analysis indicated that PPI occurred in 11 proteins; most of these were SANT domain and Wd repeat region domain-containing proteins, and were enriched in the process of protein synthesis. Important biological processes were screened by GO analysis, most of these were related to abiotic stress responses, such as “carbonate dehydratase activity” (GO:0005770), “protein targeting peroxisomes” (GO:0006625), and “glutathione peroxidase activity” (GO:0004602) (Table 2). Among these proteins, ribosomal proteins were the main node, suggesting that they might play a crucial role in the response of TtMYB1 to salt stress (Fig. 11A). Through Kyoto Encyclopedia of Genes and Genomes (KEGG) analysis, we found that 24 proteins could be annotated to known functional genes. Among them, seven proteins were enriched in “genetic information processing”, five in “signaling and cell precursors”, three in “carbohydrate metabolism”, two in “energy metabolism”, one in “environmental information processing”, and one in “cellular process” (Fig. 11B).

**Table 2:** Main GO terms of TtMYB1 interacting proteins under salt stress

Accession number	Biological process	Protein number
GO:0007031	Peroxisome organization	1
GO:0006402	mRNA catabolic process	3
GO:0031647	Regulation of protein stability	1
GO:0006625	Protein targeting to peroxisome	1
GO:0010468	Regulation of gene expression	1
GO:0008494	Translation activator activity	2
GO:0004602	Glutathione peroxidase activity	2
GO:0003729	mRNA binding	5
GO:0004089	Carbonate dehydratase activity	1
GO:0005770	Late endosome	2
GO:0032451	Demethylase activity	1

### TtMYB1 and TtHHO5 interaction identified by bimolecular fluorescence complementation (BiFC) assays

**Fig. 12:**
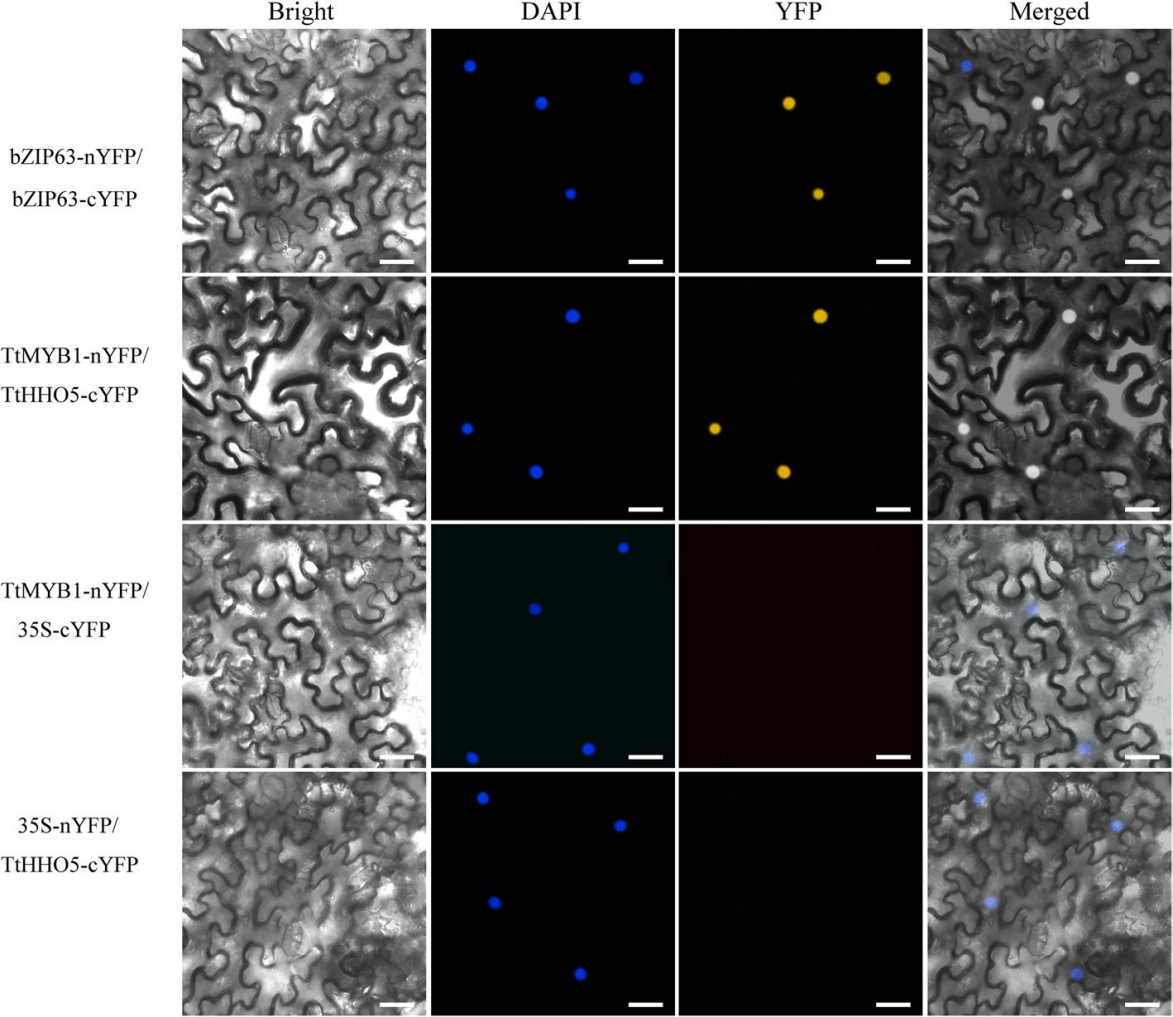
TtMYB1 interacts with TtHHO5 in *N. benthamiana* cells as measured by bimolecular fluorescence complementation (BiFC) assays. TtMYB1 was fused to the N-terminal fragment of yellow fluorescent protein (TtMYB1-nYFP), and TtHHO5 was fused to the C-terminal fragment of YFP (TtHHO5-cYFP). Co-localization of reconstituted YFP and nuclei was identified by 4′,6-diamidino-2-phenylindole (DAPI) staining. Scale bars, 25 μm

An interesting interacting transcription factor TtHHO5 from wheat was identified in plant cells by the BiFC assay. When TtMYB1-nYFP (the N-terminal fragment of yellow fluorescent protein (YFP) was transiently co-expressed with TtHHO5-cYFP (the C-terminal fragment of YFP) in *Nicotiana benthamiana* leaves, reconstituted YFP fluorescence was observed in the nuclei, indicating an interaction between TtMYB1 and TtHHO5. However, no yellow fluorescent signal was observed when TtMYB1-nYFP and cYFP or TtHHO5-cYFP and nYFP were co-expressed (Fig. 12). Meanwhile, the proteins TtMYB1 and TtHHO5 were co-localized in blue nuclei, which coincided with their regulatory function.

## Discussion

### Cloning and expression characteristics of the *TtMYB1* gene

*MYB* genes are closely associated with abiotic stress tolerance, such as salinity stress, drought stress, and high temperature [10]. Most of the R2R3-MYB genes and a few *MYBR* and *3R-MYB* genes are known to be involved in salt stress regulation. Overexpression of the wheat *TaMYB73* gene in *A. thaliana* enhances salt tolerance of transgenic plants and up-regulates the expression of several genes such as *AtCBF3*, *AtABF3*, *AtRD29A*, and *AtRD29B* [33]. Rice *OsMYB91* is an R2R3-MYB gene that is involved in tolerance to salt stress and plant growth by enhancing the ability to scavenge ROS [15]. In this study, a unique *TtMYB1* gene, which was upregulated under salt stress, was cloned from salt-tolerant *Tritipyrum* “Y1805”. Amino acid sequences indicate that TtMYB1 contains a 2R3R domain (Fig. 3). TtMYB1 is a hydrophilic protein that is rich in leucine and glycine. The model has a high number of residues (92.60%) within the favored regions of the Ramachandran plot, making it a reliable 3D protein structure. Phylogenetic analysis of MYB proteins from various species showed that TtMYB1 had a close genetic relationship with those of *Th. elongatum*, indicating that TtMYB1 should originate from the E genome of *Th. elongatum*.

Salt tolerance is attributed to the significantly induced expression of abiotic stress-response genes [34]. *MdMYB108L* overexpression also increased the photosynthetic capacity of hairy root tissue (leaves) under salt stress. In addition, the MdMYB108L TF bound to the *MdNHX1* promoter positively regulates the transcription of the salt tolerance gene *MdNHX1* in apples, improving the salt tolerance of transgenic plants [17]. Here, the relative expression level of the *TtMYB1*gene was the highest in the roots of “Y1805” under salt stress, stems were the next highest, and then leaves (Fig. 1A). The root system was directly and seriously damaged under salt stress. The *TtMYB1* expression level in the roots was significantly higher than that in the control under salt stress. However, it dropped rapidly to the control level after recovery (Fig. 1B). Therefore, *TtMYB1* was expressed highly and sensitively in the roots to adapt to salt stress.

### Interacting proteins of TtMYB1

The MYB protein family is a large, functionally diverse family that is widely involved in plant biotic and abiotic stress responses [10]. It is related to various biological processes, such as the responses to adversity stress such as salt stress, drought, and low temperature [35–37]. In GO analysis, *MYB* genes that are differentially expressed in rice were found to be involved in the regulation of plant biological processes, such as responses to abiotic stress, endogenous stimuli, environmental stimuli, and regulation of two-component signal transduction systems [38, 39]. *Arabidopsis MYB*s are related to “lipid and energy metabolism”, “osmoprotection”, “terpenoid and flavonoid metabolism”, and “signal transduction” [40]. In this experiment, TtMYB1 interacting proteins are enriched in “genetic information processing”, “environmental information processing”, “carbohydrate metabolism”, “signaling and cellular process”, and “energy metabolism” pathways, which is consistent with previous results [38, 40, 41]. MYB7 participates in salt stress during seed germination through the negative regulation of the bZIP TF ABI5 [42]. In addition to flavonoid biosynthesis and ROS scavenging, overexpression of *MYB12* also upregulated the expression of genes involved in ABA and proline biosynthesis, thus conferring plants with salt tolerance [43]. This experiment found new proteins that interacted with TtMYB1, such as the proteins related to vitamin D receptor proteins (XM_020340878.3 and XM_044495777.1). These proteins might contribute to salt tolerance in *Tritipyrum* “Y1805” (Table S2). In addition, PPI analysis showed that ribosomal proteins were the main node, suggesting that they might participate in the efficient reorganization of protein biosynthetic machinery [44], which could be attributed to the salt tolerance of the *TtMYB1* gene.

The HHO TF family is a new subfamily of the G2-like family in the GARP superfamily [45]. MYB4 had a strong regulatory influence on HHO5 under salt stress in wheat [46]. Expression of *HHO5* increases in the first 6–12 h of salt stress in *Arabidopsis*, and it is most highly expressed in vegetative tissues, predominantly roots [47]. Here, TtMYB1 and TaHHO5 showed an interaction by the Y2H and BiFC assays. It was speculated that TtMYB1 might have a regulatory effect on TtHHO5 in enhancing salt tolerance of the overexpression lines.

### Effect of *TtMYB1* gene on salt tolerance of transgenic wheat

The expression of some MYB genes is induced or inhibited by abiotic stress, which can increase the salt tolerance of transgenic plants [48, 49]. Similarly, our transcriptome results indicated that salinity stress could rapidly improve *TtMYB1* gene expression, suggesting that *TtMYB*1 might play a critical role in the salinity stress response. Therefore, we transformed *TtMYB1* into common wheat “1718” to analyze its tolerance to salt stress. Our finding suggested that the *TtMYB1* overexpression lines had less leaf withering and longer roots compared to the WT plants (Figs. 7 and 8). *TtMYB1* was similar to *Tel2E01G633100* according to their cDNA sequences, so it was speculated that they could have a similarity in function. *Tel2E01G633100* was assigned to the gibberellic acid mediated signaling pathway (GO:0009740) and gibberellin biosynthetic process (GO:0009686) by GO analysis. Gibberellin can promote the elongation and growth of roots for better water uptake under salt stress, which might be part of the reason for longer roots in “Y1805”. When plants are subjected to drought and salinity stresses, some physiological indices can react quickly to enable these plants to survive under extreme environmental conditions [48, 50]. Under high salt stress conditions, the ROS and MDA content in the transgenic *FtMYB13* plants decreased, whereas proline content and photosynthetic efficiency increased, which improved their salt tolerance [31]. Physiological indices related to osmotic stress caused by drought and salinity can be used as a fast and accurate method for assessing plant resistance to abiotic stress [50–52]. Abiotic stress has been reported to cause lipid peroxidation, leading to MDA accumulation [50, 53]. Here, the MDA content was higher in WT plants than in *TtMYB1* overexpression lines (Fig. 9C), which indicated that salt stress damaged WT plants more than the overexpression lines. Therefore, the *TtMYB1* gene might contribute to the cell membrane integrity of plants in response to salinity stress. In response to salt stress, proline and soluble sugar participate in osmotic regulation and maintain the structural stability of cells [54]. In this study, the overexpression lines accumulated more proline and soluble sugar compared with the WT plants under salt stress (Fig. 9A, B), which reduced the water potential of root cells, increased the water absorption and retention capacity of roots, and finally improved their salt tolerance. Therefore, our results demonstrate that salt tolerance of the *TtMYB1* overexpression lines was partially related to higher proline and soluble sugar content, and lower levels of MDA.

## Conclusion

A novel *TtMYB1* gene was selected from salt-tolerant *Tritipyrum* “Y1805” based on transcriptome data. The *TtMYB1* gene had a coding sequence length of 783 bp and had 95.79% identity to *Tel2E01G633100* from *Th. elongatum*. A phylogenetic tree showed that TtMYB1 and MYB from *Th. elongatum* were clustered on the same branch. The *TtMYB1* gene was significantly upregulated in “Y1805” roots to adapt to salt stress. The *TtMYB1* gene was located in the nucleus. Subsequently, the *TtMYB1* overexpression vector was transformed into common wheat. Under NaCl stress, the leaves of the *TtMYB1* overexpression lines showed less wilting, and greater root length and seedling height than those of WT plants. In addition, compared to WT plants under salt stress, the overexpression lines had significantly higher proline and soluble sugar content, but lower MDA content. Overall, 51 proteins interacting with TtMYB1 were identified by Y2H. Through PPI analysis, most of the proteins were SANT and Wd repeats region domain-containing proteins, and ribosomal proteins were the main node. Abiotic stress-related terms were enriched in GO analysis. KEGG analysis indicated that “genetic information processing”, “signaling and cell precursors”, “carbohydrate metabolism”, and “environmental information processing” pathways were the most enriched. Thus, the overexpression of the *TtMYB1* gene could enhance salt tolerance in wheat and might be a valuable gene for improving crop salt tolerance.

## Materials and methods

### Plant materials

The materials are salt-tolerant octoploid *Tritipyrum* (“Y1805”, AABBDDEE) and salt-sensitive common wheat (AABBDD) “CS”, and “1718”. *Tritipyrum* “Y1805” is a stable progeny from a wide cross between *Triticum aestivum* and *Th. elongatum*. “Y1805” contains A, B, and D chromosomes of wheat, as well as a group of E chromosomes of *Th. Elongatum* [55]. “Y1805” is deposited in Guizhou Subcenter of National Wheat Improvement Center (Guiyang, China). *TtMYB1* was transformed into salt-sensitive common wheat “1718” for salt-tolerant identification.

### Plant growth conditions and stress treatments

The seeds of *Tritipyrum* “Y1805” and wheat “CS” were germinated in a growth chamber (relative humidity of 75% and a temperature of 20/15°C, light/dark). The seedlings were sown on a floater board in 1/2 Hoagland’s solution with a 16/8 h light/dark cycle, an irradiance of 400 µmol m^−2^s^−1^, and the same temperature and humidity as in the germination chamber. The culture solution was refreshed every 3 days. At the two-leaf stage (the 14th day after germination), salt stress treatments (1/2 Hoagland’s solution added with 250 mM NaCl) were started. The first wheat roots, stems, and leaves of uniform size were selected at 5 h after salt stress according to the preliminary experiments. The materials were recovered in 1/2 Hoagland’s solution without NaCl after 24 h of salt stress. The second samplings were performed at 1 h after recovery. Normal (1/2 Hoagland’s solution without NaCl) cultured materials were used as the controls. All tissue samples were immediately frozen in liquid nitrogen after sampling and stored at − 80 °C for qPCR analysis and gene cloning. The experiment was conducted with three biological replications, and each replication contained at least 10 seedlings.

### Screening of differentially expressed genes (DEGs)

Previous RNA-seq raw reads were deposited into the NCBI SRA database under accession no. PRJNA769794. The DEG screening was based on a method described previously [55]. The DEGs potentially regulated by the treatments were identified based on a false discovery rate (FDR) threshold of < 0.01, *p* < 0.001, and absolute log_2_fold change value (|log_2_FC|) > 1 of the three salt-treated and three salt-free samples using DESeq software [56]. The Phyper function in the R package was used for the enrichment analysis of GO categories.

### Expression pattern analysis by qPCR

Total RNA from the tissues of roots, stems, and leaves was reverse-transcribed using Power SYBR Green PCR Master Mix (Applied Biosystems, Foster City, CA, USA). Amplification by qPCR was performed on an ABI StepOne Real-Time PCR System. The relative expression levels were calculated using the 2^−ΔΔ Ct^ method, with three biological replications and three technical replications [57], and *18 S* RNA was used as the internal control.

### RNA reverse transcription, *TtMYB1* amplification and plasmid construction

RNA was reverse-transcribed into cDNA using a PrimeScript RT kit (Takara, Dalian, China). The full-length coding sequence of *TtMYB1* was amplified from the cDNA of “Y1805” using primers containing a BsaI restriction site at the 5′ and 3′ ends of the amplified fragment. The amplification primers are listed in Table S3. The pEGOEPubi-H-*TtMYB1*-GFP vector was constructed using T4-DNA ligase (Takara) following the manufacturer’s protocol. This vector has been modified to contain the *GFP* gene. The inserted sequence was driven by a corn UBI promoter.

### Bioinformatics analysis of the *TtMYB1* gene

Multiple alignment of *TtMYB1* sequences was performed using DNAMAN with the complete alignment method. Displaying complete base sequence and coloring 100% of homologous bases were selected as the parameters. Online tools Expasy for hydropathy prediction, and NetPhos 2.0 and CPHmodels 3.2 for phosphorylation site analysis were adopted. The homologous sequences of the MYB protein were retrieved by a BLAST search in the NCBI database (accession numbers in Table S4). A phylogenetic tree was constructed using MEGA 7 (Mega Limited, Auckland, New Zealand) with the maximum-likelihood method and 1000 bootstraps.

Homology modeling of the TtMYB1 protein was performed using MODELLER 9.22 [58]. The X-ray crystal structure of a putative MYB protein At2g46140.1 (PDB ID: brp8) was utilized as a template for this procedure. SAVES was used to analyze the predicted model. GROMACS software was used to calculate the RMSD and potential energy value of the model protein [59]. Ramachandran plots were analyzed using the Rampage server [60].

### Subcellular localization of TtMYB1

The construct containing TtMYB1 was transiently transformed into mesophyll cells prepared from the leaves of 4-week-old tobacco with a polyethylene glycol-mediated protocol. The transformed mesophyll cells were incubated for 72 h at 22 °C in the dark. GFP fluorescence was observed in transformed tobacco mesophyll cells using a confocal laser-scanning microscope (FV1000, Olympus, Tokyo, Japan).

### Transformation and detection of *TtMYB1*

*TtMYB1* was transformed into common wheat “1718” according to the wheat coleoptile method [61]. For the selection of transformants, seedlings of T_0_ transgenic wheat containing the GFP reporter gene were detected using a LUYOR-3415RG hand-held lamp (LUYOR Corporation, Shanghai, China), and planted in a field. Their leaves were sampled at the seedling stage for further PCR identification of transgenic plants. Putative transformants were tested by multiplex PCR amplification of genomic DNA using the specific primers for the UBI promoter and GFP gene, and the primers for the *18 S* housekeeping gene (Table S3). PCR products were separated on 1% (w/v) agarose gel.

### Phenotype and physiological determination of transgenic wheat

Homozygous T_3_ transgenic wheat lines were selected for the assays of growth (root length and seedling height) and physiological analysis. Samples were collected at 0 h (control), 5 h (T1 stage), and 24 h (T2 stage) after salt stress, and at 1 h (R stage) after recovery. The assay kits were applied for measuring proline content (Cas no.: BC0295, Solarbio, Beijing, China), soluble sugar (Cas no. BC0035, Solarbio), and MDA (Cas no.: BC0025, Solarbio) in accordance with the manufacturer’s instructions. Three biological replications were performed, and each replication contained at least 10 seedlings for the phenotype and physiological determination in this experiment.

### Y2H screen of TtMYB1 interacting proteins

The cDNA library was constructed and identified according to Yang’s method [61]. The full-length coding sequence of *TtMYB1* was inserted into the bait vector pGBKT7 (Takara). The recombinant construct was introduced into the yeast strain Y2HGold using the polyethylene glycol/LiAc method and tested for autoactivation and toxicity. SD-LW and SD-ALWH plates (Coolaber, Beijing, China) were used to screen monoclonal colonies (> 2 mm), and selected colonies were placed in SD-LW liquid medium with shaking during the logarithmic growth phase (29 °C, 200 rpm, 20 h) and then plated on SD-ALWH medium. Positive clones were selected on SD-LW and SD-ALWH (Coolaber) media. The sequencing results of positive clones were analyzed via BLAST.

### Switch back prey plasmid and confirmation of positive interactions in yeast

The selected positive clones were extracted using a yeast plasmid extraction kit (Solarbio) and then transformed into *E. coli DH5α* (Collaborative Innovation Center for the Prevention and Control of Infectious Diseases in the Western Region, Xi’an, China). After culturing, the plasmid was extracted with a plasmid extraction kit (Tiangen, Beijing, China), and the plasmid and pGBKT7-TtMYB1 were co-transformed into yeast Y2HGold cells. The positive cells were screened by coating SD-LW, SD-ALWH, and SD-ALWH/(x-α-gal) auxotrophic plates. GO and KEGG enrichment analyses and PPI analysis were performed for these interacting proteins [62–64].

### BiFC assays

The BiFC assays were conducted as previously described [65]. The full-length cDNAs of *TtMYB1* and *TtHHO5* were cloned into the vectors of pUC-SPYNE (N terminus of YFP) and pUC-SPYCE (C terminus of YFP) to obtain TtMYB1-nYFP and TtHHO5-cYFP, and then transformed into *Agrobacterium* (line GV3101). Two vector combinations were co-transformed into 2-4-week-old *N. benthamiana* leaves. At 72 h after incubation, YFP signals were imaged by confocal laser microscopy (FV1000, Olympus). Nuclei were stained with DAPI (Sigma-Aldrich, St. Louis, USA). Three biological repeats were included for this experiment.

### Statistical analyses

Statistical software (SPSS 20.00, IBM Inc., Armonk, NY, USA) and graphics software (Origin 2017, OriginLab Inc., Northampton, MA, USA) were used for the data analysis and figure construction, respectively. Duncan’s multiple range test was performed to determine significant differences between means at a significance level of *p* < 0.05 after displaying a significant effect during an ANOVA analysis.

### Supplementary Information

**Additional file 1: Supplementary Fig. S1.** The original gel image of Figure 2A. Amplified band with “Y1805” cDNA as a template. 1, 2: Amplified bands; M, 2000 bp DNA marker.

**Additional file 2: Supplementary Fig. S2.** The original gel image of Figure 6. PCR detection of the *TtMYB1* gene in the genomic DNA of putative transgenic T_0_ wheat leaves. The fragment of the amplified *TtMYB1* gene is indicated by a yellow arrow. A white arrow shows the band of the amplified housekeeping *18S* gene. M, 2,000 bp DNA marker; lanes 1, 5, 8, 10, and 12, transgenic plants; lanes 2, 3, 4, 6, 7, 9, and 11, non-transformed plants; P, positive control (*TtMYB1* recombinant plasmid); N, negative control (wild-type DNA).

**Additional file 3: Table S1.** The sequence of truncated* TtMYB1 *for self-activation detection. **Table S2. **Sequencing data and prediction results of potential interacting proteins with TtMYB1. **Table S3.** The primers used in this study. **Table S4.** The proteins in *Gramineae* plants.

## Data Availability

All data generated or analyzed for this study are included in this article and its supplementary files.
