# Supplementary figures and images for: Characterization and transformation of TtMYB1 transcription factor from Tritipyrum to improve salt tolerance in wheat
Source: BMC Genomics. 2024 Feb 9;25:163. doi: 10.1186/s12864-024-10051-5 (PMC10854188; doi:10.1186/s12864-024-10051-5)

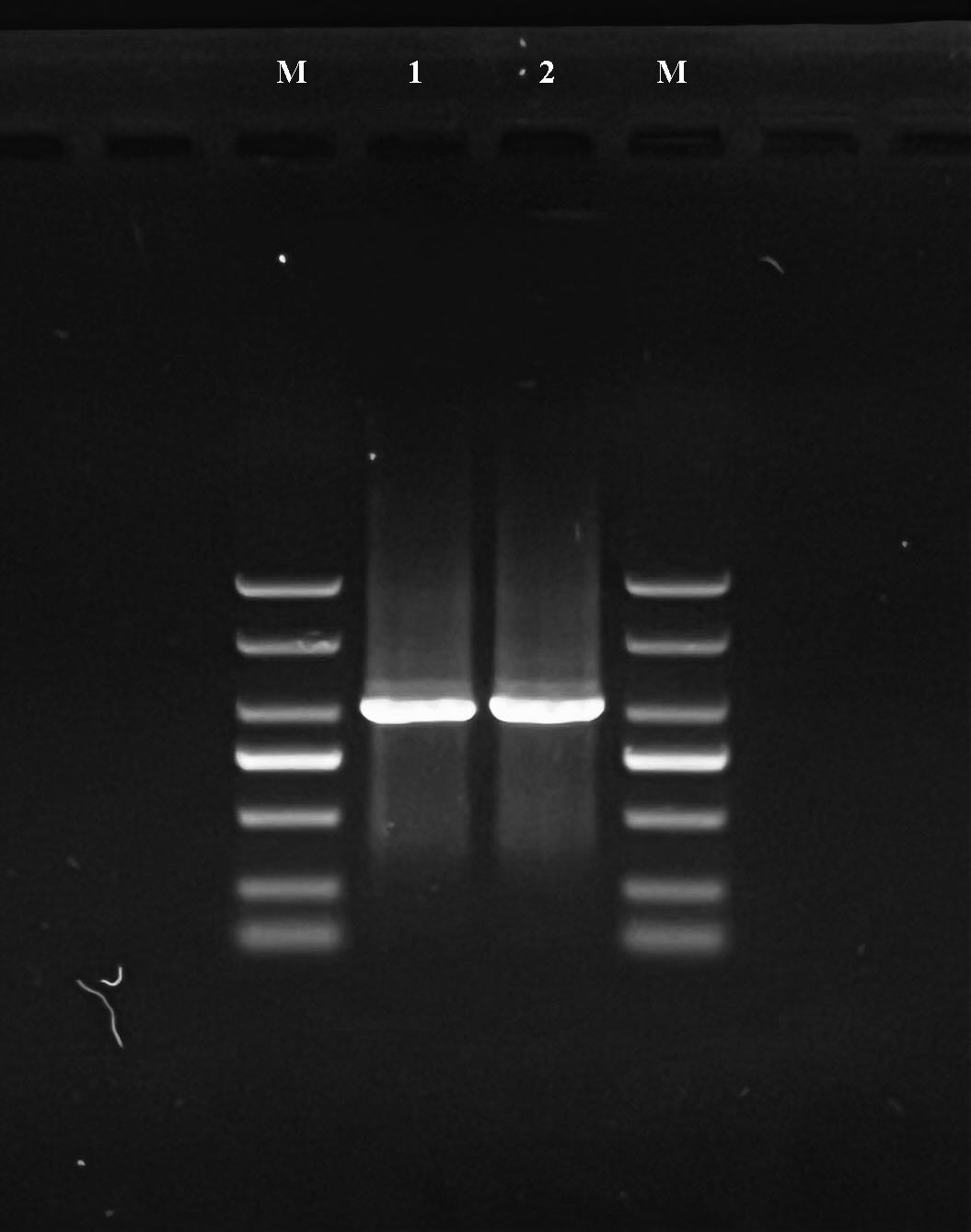

Supplement: Supplementary file 1 — Additional file 1: Supplementary Fig. S1. The original gel image of Figure 2A. Amplified band with “Y1805” cDNA as a template. 1, 2: Amplified bands; M, 2000 bp DNA marker. [file 12864_2024_10051_MOESM1_ESM.jpg]

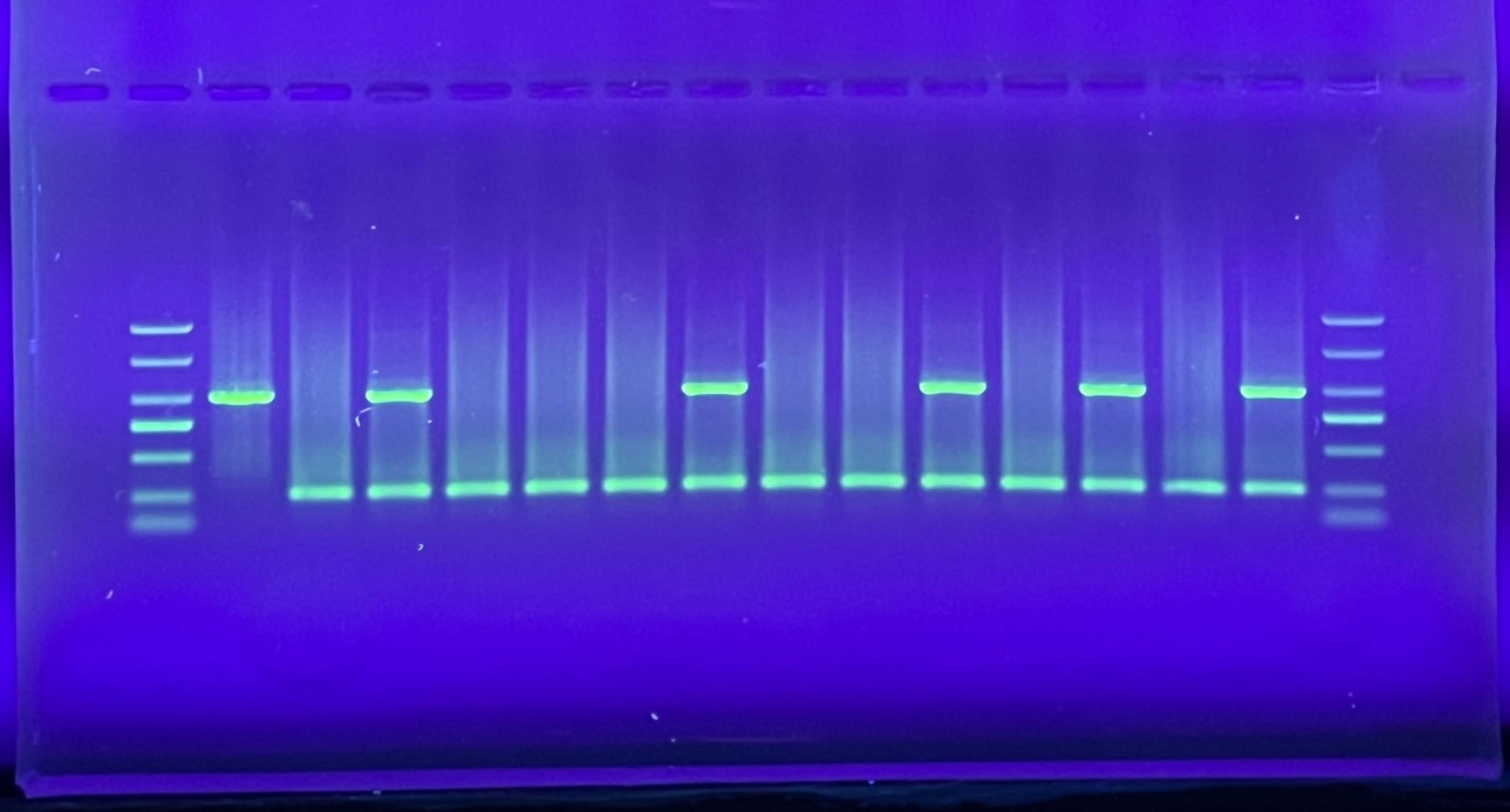

Supplement: Supplementary file 2 — Additional file 2: Supplementary Fig. S2. The original gel image of Figure 6. PCR detection of the TtMYB1 gene in the genomic DNA of putative transgenic T0 wheat leaves. The fragment of the amplified TtMYB1 gene is indicated by a yellow arrow. A white arrow shows the band of the amplified housekeeping 18S gene. M, 2,000 bp DNA marker; lanes 1, 5, 8, 10, and 12, transgenic plants; lanes 2, 3, 4, 6, 7, 9, and 11, non-transformed plants; P, positive control (TtMYB1 recombinant plasmid); N, negative control (wild-type DNA). [file 12864_2024_10051_MOESM2_ESM.jpg]
