# Supplementary material for: Characterization and transformation of TtMYB1 transcription factor from Tritipyrum to improve salt tolerance in wheat
Source: BMC Genomics. 2024 Feb 9;25:163. doi: 10.1186/s12864-024-10051-5 (PMC10854188; doi:10.1186/s12864-024-10051-5)
Supplement: Supplementary file 3 — Additional file 3: Table S1. The sequence of truncated TtMYB1 for self-activation detection. Table S2. Sequencing data and prediction results of potential interacting proteins with TtMYB1. Table S3. The primers used in this study. Table S4. The proteins in Gramineae plants. [file 12864_2024_10051_MOESM3_ESM.docx]

**Table S1.** The sequence of truncated *TtMYB1* for self-activation detection.

| *TtMYB* fragments | The sequence of *TtMYB* fragments |
| --- | --- |
| *TtMYB1-1* | ATGGCGGAGGCGGAAGGGCAGCTCGCGGCCTGCTGGGGGAAGCAGGACGACGAGTGGAGGAAAGGCCCGTGGACAACCCAGGAGGACAAGCTGCTCCTCGACCATGTCGCCCAGCACGGCGAAGGGAGGTGGAACTCCGTCTCCAAGCTCACAGGTCTCAAGAGAAGTGGCAAGAGTTGCAGGCTGCGGTGGGTTAACTACCTCAGACCTGATCTAAAGAGAGGCAAGATGACACCCCAAGAGGAGAGCACCATAGTCCAGCTCCACTCCTTGTGGGGGAACAGGTGGTCGACGATTGCACGCAGCCTCCCGGGGAGGACGGACAACGAGATCAAGAACTACTGGAGGACGCACTACAAGAAG |
| *TtMYB1-2* | GGCAAGCCGTCCAAGAACATCGAGCGCGCCAGGGCTCGGTTCCTGATGCAGCGGCGTGAGATGCAGCAGCAGCAGCAGCAGCAGCAACAGAAGCTGCTACTTGGGCAGGGCAAGGACGCCGAGCTCGCCAGCGCCACTGCCGAGGACGACGTCGACGAGAAGATGACCGGCAACGTAGGCCCGGCAGCGACGGCGCTGGCTGACCACGGCCACGAGGAGCTGATCATGCAGGACGTCTTGGACTTCCTGTGCCCCATGTCCTGCGCCCTCCTCCACAGCGCCGGGCAGAGCGGCAGCTGCGGCGCCTCCACGAGCGAGGAGTATGGGTCGACCGAGGATGACGGCGCCACGTGGGGAAGCCTGTGGAACCTCGAAGACGTGGCCCATGACGGCGACGGTGGGGCATGCGCGCTGTGGTAG |

**Table S2.** Sequencing data and prediction results of potential interacting proteins with TtMYB1.

| No. | Sequence ID | Predicted result by BLAST analysis | Identities | |
| --- | --- | --- | --- | --- |
| 1 | [XM_045115069.1](https://www.ncbi.nlm.nih.gov/nucleotide/XM_045115069.1?report=genbank&log$=nuclalign&blast_rank=1&RID=VYW61RDT016) | *Hordeum vulgare subsp*. vulgare probable inactive carboxylesterase Os04g0669700 (LOC123431245), mRNA | 156/170(92%) | |
| 2 |  | No significant similarity found. |  |  |
| 3 | XM_044520006.1 | *Triticum aestivum* RNA demethylase ALKBH10B-like (LOC123098115), transcript variant X2, mRNA | 473/474(99%) | |
| 4 | XM_037629064.1 | *Triticum dicoccoides* glycine-rich RNA-binding protein RZ1C-like (LOC119363694), mRNA | 405/406(99%) | |
| 5 | EF534375.1 | intermedium SGT1 cDNA, complete cds | 872/879(99%) | |
| 6 |  | No significant similarity found. |  |  |
| 7 | XM_044570403.1 | *Triticum aestivum* uncharacterized protein At2g39795, mitochondrial-like (LOC123150563), mRNA | 596/596(100%) | |
| 8 | XM_044551351.1 | *Triticum aestivum* protein KINESIN LIGHT CHAIN-RELATED 1-like (LOC123131681), mRNA | 881/883(99%) | |
| 9 | XM_044527128.1 | *Triticum aestivum* transcription factor HHO5 (LOC100873121), mRNA | 793/795(99%) | |
| 10 | XM_044548359.1 | *Triticum aestivum* transcription factor TCP14-like (LOC123128376), mRNA | 866/881(98%) | |
| 11 | XM_020325097.3 | *Aegilops tauschii subsp. strangulata* calnexin homolog (LOC109766335), mRNA | 882/882(100%) | |
| 12 | XM_044551351.1 | *Triticum aestivum* protein KINESIN LIGHT CHAIN-RELATED 1-like (LOC123131681), mRNA | 910/916(99%) | |
| 13 | XM_044519104.1 | *Triticum aestivum* probable glutathione peroxidase 4 (LOC123097394), mRNA | 878/884(99%) | |
| 14 | XM_044469297.1 | *Triticum aestivum* vesicle-associated protein 1-2-like (LOC123046019), transcript variant X1, mRNA | 881/885(99%) | |
| 15 | XM_044583951.1 | *Triticum aestivum* BTB/POZ domain-containing protein NPY1-like (LOC123166180), transcript variant X2, mRNA | 909/911(99%) | |
| 16 | XM_044490749.1 | *Triticum aestivum* peroxisomal membrane protein PEX14-like (LOC123068242), transcript variant X1, mRNA | 878/880(99%) | |
| 17 | XR_006465824.1 | T*riticum aestivum* uncharacterized LOC123135110 (LOC123135110), ncRNA | 410/477(86%) | |
| 18 | XM_044597708.1 | *Triticum aestivum* uncharacterized LOC123185884 (LOC123185884), transcript variant X2, mRNA | 878/882(99%) | |
| 19 | XM_044574765.1 | *Triticum aestivum* fructokinase-2-like (LOC123156616), mRNA | 877/884(99%) | |
| 20 | XM_044557843.1 | *Triticum aestivum* ATP synthase subunit O, mitochondrial-like (LOC123137946), mRNA | 874/922(95%) | |
| 21 | XM_044594237.1 | *Triticum aestivum* alpha-glucosidase 2-like (LOC123181840), mRNA | 884/884(100%) | |
| 22 | XM_020340878.3 | *Aegilops tauschii subsp*. strangulata protein SRC2 (LOC109782277), mRNA | 824/894(92%) | |
| 23 | XM_044468152.1 | *Triticum aestivum* LOB domain-containing protein 40-like (LOC123045197), mRNA | 549/549(100%) | |
| 24 | XM_020311993.3 | *Aegilops tauschii subsp*. strangulata alpha-glucosidase 2 (LOC109753062), mRNA | 824/859(96%) | |
| 25 | XM_044495777.1 | *Triticum aestivum* protein SRC2-like (LOC123072210), mRNA | 851/855(99%) | |
| 26 | XM_044492352.1 | *Triticum aestivum* beta-glucosidase 32-like (LOC123069478), mRNA | 882/887(99%) | |
| 27 |  | No significant similarity found. |  |  |
| 28 | XM_044495777.1 | *Triticum aestivum* protein SRC2-like (LOC123072210), mRNA | 909/918(99%) | |
| 29 | XM_044563084.1 | *Triticum aestivum* protein RRC1-like (LOC123144063), transcript variant X3, mRNA | 556/631(88%) | |
| 30 | XM_044597708.1 | *Triticum aestivum* uncharacterized LOC123185884 (LOC123185884), transcript variant X2, mRNA | 880/883(99%) | |
| 31 | XM_044511018.1 | *Triticum aestivum* purine permease 3-like (LOC123089306), mRNA | 766/794(96%) | |
| 32 | XM_037612029.1 | *Triticum dicoccoides* uncharacterized LOC119340121 (LOC119340121), mRNA | 882/883(99%) | |
| 33 | XM_020325097.3 | *Aegilops tauschii subsp*. strangulata calnexin homolog (LOC109766335), mRNA | 875/881(99%) | |
| 34 | XM_044594237.1 | *Triticum aestivum* alpha-glucosidase 2-like (LOC123181840), mRNA | 774/777(99%) | |
| 35 | XR_006426081.1 | *Triticum aestivum* polyubiquitin 11-like (LOC123054201), misc_RNA | 720/721(99%) | |
| 36 |  | No significant similarity found. |  |  |
| 37 | XM_044466667.1 | *Triticum aestivum* kinesin-like protein KIN-12E (LOC123044045), transcript variant X2, mRNA | 883/883(100%) | |
| 38 | XM_044583951.1 | *Triticum aestivum* BTB/POZ domain-containing protein NPY1-like (LOC123166180), transcript variant X2, mRNA | 878/881(99%) | |
| 39 | XM_048703375.1 | *Triticum urartu* 40S ribosomal protein S20 (LOC125539852), mRNA | 401/417(96%) | |
| 40 | XM_044466362.1 | *Triticum aestivum* vegetative cell wall protein gp1 (LOC100415857), mRNA | 824/887(93%) | |
| 41 | XM_044524872.1 | *Triticum aestivum* probable plastid-lipid-associated protein 2, chloroplastic (LOC123103328), mRNA | 883/883(100%) | |
| 42 | XM_020297570.3 | *Aegilops tauschii subsp*. strangulata gamma carbonic anhydrase 1, mitochondrial (LOC109738476), mRNA | 880/882(99%) | |
| 43 | XM_020311608.3 | *Aegilops tauschii subsp*. strangulata elongation factor 2 (LOC109752678), mRNA | 880/884(99%) | |
| 44 | XM_044594237.1 | *Triticum aestivum* alpha-glucosidase 2-like (LOC123181840), mRNA | 774/775(99%) | |
| 45 | XM_044524872.1 | *Triticum aestivum* probable plastid-lipid-associated protein 2, chloroplastic (LOC123103328), mRNA | 882/883(99%) | |
| 46 | XM_037623634.1 | *Triticum dicoccoides* 40S ribosomal protein S20 (LOC119356651), mRNA | 498/498(100%) | |
| 47 | XM_044562603.1 | *Triticum aestivum* calnexin homolog (LOC123143657), mRNA | 850/883(96%) | |
| 48 | XM_044463388.1 | T*riticum aestivum* NAC domain-containing protein 92-like (LOC123040589), mRNA | 836/852(98%) | |
| 49 |  | No significant similarity found. |  |  |
| 50 | XR_002232991.3 | *Aegilops tauschii subsp*. strangulata uncharacterized LOC109763776 (LOC109763776), ncRNA | 909/912(99%) | |
| 51 | XM_044551351.1 | *Triticum aestivum* protein KINESIN LIGHT CHAIN-RELATED 1-like (LOC123131681), mRNA | 879/882(99%) | |

**Table S3.** The primers used in this study.

| Primer role | Primer sequence (5'-3') |
| --- | --- |
| *TtMYB1* amplification | Actag ggtctcG cacc ATGGCGGAGGCGGAAGGGCA  Actag ggtctcT cgcc CCACAGCGCGCATGCCCCAC |
| PCR identification of transformant | TTAGCCCTGCCTTCATACGC  GACACGCTGAACTTGTGG |
| *18S* | TCGGGATCGGAGTAATGA  TTCGCAGTTGTTCGTCTT |

**Table S4.** The proteins in *Gramineae* plants.

| Species | Protein ID |
| --- | --- |
| *Thinopyrum elongatum* | Tel2E01G633100 |
| *Thinopyrum elongatum* | CM022298.1 |
| *Hordeum vulgare* | XP 044970301.1 |
| *Triticum urartu* | XP 048553034.1 |
| *Triticum aestivum* L. | XP 044320143.1 |
| *Triticum dicoccoides* | XP 037486315.1 |
| *Aegilops tauschii* | XP 020160476.1 |
| *Sorghum bicolor* | XP 021319701.1 |
| *Zea mays* L. | XP 008645936.1 |
| *Oryza sativa* L. | XP 015624171.1 |
